# Pharmacokinetics of tulathromycin in pregnant ewes (*Ovis aries*) challenged with *Campylobacter jejuni*

**DOI:** 10.1371/journal.pone.0256862

**Published:** 2021-08-27

**Authors:** Michael Yaeger, Jonathan P. Mochel, Zuowei Wu, Paul Plummer, Orhan Sahin, Joseph Smith, Melda Ocal, Ashenafi Beyi, Changyun Xu, Qijing Zhang, Ronald W. Griffith

**Affiliations:** 1 Department of Veterinary Pathology, College of Veterinary Medicine, Iowa State University, Ames, Iowa, United States of America; 2 Department of Veterinary Diagnostic & Production Animal Medicine, College of Veterinary Medicine, Iowa State University, Ames, Iowa, United States of America; 3 Department of Biomedical Sciences, College of Veterinary Medicine, Iowa State University, Ames, IA, United States of America; 4 Department of Veterinary Microbiology and Preventative Medicine, College of Veterinary Medicine, Iowa State University, Ames, IA, United States of America; 5 Department of Large Animal Clinical Sciences, College of Veterinary Medicine, University of Tennessee, Knoxville, TN, United States of America; Bahauddin Zakariya University, PAKISTAN

## Abstract

The purpose of this study was to evaluate the pharmacokinetics of tulathromycin in the plasma and maternal and fetal tissues of pregnant ewes when administered within 24 hours of a single, IV *Campylobacter jejuni* (*C*. *jejuni*) challenge. Twelve, pregnant ewes between 72–92 days of gestation were challenged IV with *C*. *jejuni* IA3902 and then treated with 1.1 ml/45.36 kg of tulathromycin subcutaneously 18 hours post-challenge. Ewes were bled at predetermined time points and euthanized either at a predetermined time point or following the observation of vaginal bleeding or abortion. Following euthanasia, tissues were collected for bacterial culture, pharmacokinetics and histologic examination. The maximum (geometric) mean tulathromycin plasma concentration was estimated at 0.302 μg/mL, with a peak level observed at around 1.2 hours. The apparent systemic clearance of tulathromycin was estimated at 16.6 L/h (or 0.28 L/kg/h) with an elimination half-life estimated at approximately 22 hours. The mean tissue concentrations were highest in the uterus (2.464 μg/g) and placentome (0.484 μg/g), and were lowest in fetal liver (0.11 μg/g) and fetal lung (0.03 μg/g). Compared to previous reports, results of this study demonstrate that prior IV administration of *C*. *jejuni* appeared to substantially alter the pharmacokinetics of tulathromycin, reducing both the peak plasma concentrations and elimination half-life. However, additional controlled trials are required to confirm those observations.

## Introduction

Infection with *Campylobacter* spp. is one of the most common causes of ovine abortion throughout the world and has been reported to be the most common infectious cause of ovine abortion in the United States [1–3]. Currently, a hypervirulent, tetracycline resistant, *C*. *jejuni* clone, termed the Sheep Abortion (SA) clone, is responsible for 93% of ovine *C*. *jejuni* abortions in the Midwestern US [1,4]. The only antibiotic approved to treat or prevent campylobacter abortion in sheep is chlortetracycline [5]. However, the vast majority of recent US *C*. *jejuni* isolates are reported to be resistant to tetracyclines (including chlortetracycline), and pharmacokinetic studies have shown that feeding chlortetracycline to sheep at the approved dose (80 mg/head/day), or at an even higher, unapproved dose (500 mg/head/day), resulted in levels that were presumed to be subtherapeutic in pregnant ewe plasma and largely undetectable in fetal tissues and amniotic fluid [1,6–9]. Results of these studies indicate that feeding chlortetracycline is unlikely to provide therapeutic benefit during an outbreak of *C*. *jejuni* abortion in US flocks.

Susceptibility results on *C*. *jejuni* isolates from field cases of ovine abortion demonstrate that the vast majority are currently susceptible to macrolide antibiotics, including tulathromycin, azithromycin, telithromycin and erythromycin [1,8,9] The potential benefits of treating sheep with a macrolide antibiotic such as tulathromycin include the ease of administration (subcutaneous injection), its wide volume of distribution, low effective plasma concentrations, and long terminal half-life, which has been reported to be 110.8 h (± 20.9) in pregnant ewes [10,11]. Pharmacokinetic studies in pregnant ewes have demonstrated that tulathromycin reaches detectable levels in fetal plasma and amniotic fluid that persist for days following administration to the ewe [11]. The potential clinical effectiveness of tulathromycin in *C*. *jejuni* exposed pregnant ewes has been confirmed in a study utilizing an IV *C*. *jejuni* challenge model in which tulathromycin treatment resulted in a statistically significant (*p* < .05) decrease in the rate of vaginal bleeding/abortion in pregnant ewes compared to untreated controls [12].

The plasma pharmacokinetics of tulathromycin have been studied in non-gravid ewes and healthy pregnant sheep [11,13]. However, these studies did not evaluate the pharmacokinetics in several tissues targeted by campylobacter during the abortion process, including the uterus and placenta/placentome. It is also well recognized that disease states can alter drug pharmacokinetics [14–17]. Pathological states may affect the binding of drugs to plasma proteins, circulatory changes can impede or enhance drug entry to specific tissues, and many diseases can alter hepatic and/or renal clearance [14]. For these reasons it is important to assess the pharmacokinetics of an antibiotic in the target tissues of diseased animals to fully appreciate their efficacy for a specific disorder.

Field and experimental studies of campylobacter abortion in sheep have consistently identify large numbers of the organism in the maternal uterus, fetal placenta and lung, and to a lesser extent fetal liver [12]. It is currently unknown how tulathromycin accumulates in these tissues in ewes undergoing infection with *C*. *jejuni*. The objective of this study was to evaluate the pharmacokinetics of subcutaneously administered tulathromycin in the plasma of ewes challenged with a single IV dose of *C*. *jejuni*. An additional objective of this investigation was to evaluate the concentrations of tulathromycin in ewe uterine and placental tissues, as well as in fetal lung, liver, and amniotic fluid in ewes challenged with *C*. *jejuni*.

## Materials and methods

### Animals

Twelve, timed-bred pregnant ewe-lambs (Hampshire ram crossed to Polypay ewe-lmbs) were sourced from the Iowa State University Sheep Teaching facility. Ultrasound was used to confirm pregnancy in all animals and to estimate the stage of gestation. Gestational ages on arrival were estimated to vary from 72–92 days of gestation. The weight of ewe-lambs ranged from 45.9–75.3 kg, with a mean weight of 58.5 kg. Ewes were allowed to acclimate for 3 days upon arrival at the Laboratory Animal Resources (LAR) research facility. Ewes were weighed on entry, identified with ear tags and were housed in pairs in pens on raised tenderfoot decks in an animal biosafety level 2 facility. All ewes were fed Teklad-Envigo 7060 small ruminant complete ration and water *ad libitum*. All undertaken procedures were approved by the Iowa State University Institutional Animal Care and Use Committee (IACUC-18-1134, protocol 7-12-7407-OP). For all inoculations, ewes were fully conscious and restrained manually.

### *Campylobacter* strain

All 12 ewes were challenged with *Campylobacter jejuni* IA3902, which is a clinical isolate of the Sheep Abortion (SA) clone cultured from an aborted ovine fetus. Although currently there is no CLSI-derived breakpoint for tulathromycin against C. jejuni in sheep, this isolate was considered susceptible based on agar dilution studies producing an MIC of 0.5 μg/mL, which is much lower than comparable breakpoints for other macrolides. This isolate was susceptible to tulathromycin based on broth microdilution and confirmed to be a SA clone by pulsed-field gel electrophoresis, multilocus sequence typing, *cmp* gene sequence typing, and whole genome sequencing [1,18]. Fresh bacterial cultures were obtained following 24 hours of growth on Mueller Hinton (MH) agar in anaerobic jars under microaerobic conditions (5% oxygen, 10% carbon dioxide, and 85% nitrogen) at 42°C. *Campylobacter* was harvested from MH agar, washed once with PBS to remove free endotoxin, diluted to the desired concentration in sterile PBS based on optical density (OD600 = 0.507), and then used as the inoculum for IV challenge. The final number of organisms in each suspension was determined by counting the number of viable CFUs.

### *C*. *jejuni* challenge

The upper half of the left jugular furrow was shaved. The area was aseptically prepared using alternate Chlorhexidine^®^ (chlorhexidine gluconate 2.0%) and isopropyl alcohol (70%) wipes performed three times. An 18G, 2-inch IV catheter was placed into the left jugular vein. Ewes were administered 1.5 ml of 50 mg/ml flunixin meglumine (Prevail) IV to lessen the impact of endotoxin. This was followed by 1–1.25 ml of 8.5X10^8^ CFU/ml *C*. *jejuni* IA3902 IV [12]. A small amount of blood was aspirated back into the syringe and reinfused following inoculation to assure that all of the inoculum was administered into the jugular vein. Because ewe weights varied from 45.9–75.3 kg, graded challenge doses were administered. Ewes weighing 45.4–54.4 kg received a 1ml challenge, ewes weighing 54.5–63.5 kg received 1.15 ml and ewes weighing >63.5 lbs received a 1.25 ml challenge dose.

### Treatment

Each ewe was administered 1.1 ml/45.36 kg of 100mg/ml (i.e., 140 ± 19 mg or 2.4 ± 0.3 mg/kg on average) tulathromycin (Draxxin, Zoetis, Parsipanny, NJ) subcutaneously in the region anterior to the axilla 18 hours post-campylobacter challenge.

### Plasma and tissue collection

Plasma was collected from each ewe at the following time points: 0 (pre-dosing), 0.5, 1, 2, 6, 12, 24, 72, 144, 216, 288, and 360 hours. Three ewes were scheduled to be euthanized on days 2, 5, 10 and 20 post-antibiotic administration for pharmacokinetics on uterus, placentome, fetal lung, liver and amniotic fluid.

### Indicators for study termination

Animals were monitored twice daily for signs of ill health including depression, loss of appetite, prolonged recumbency, elevated temperatures and evidence of impending abortion (vaginal bleeding) or abortion. Ewes that became markedly depressed and recumbent post-challenge due to endotoxemia were euthanized for humane reasons. Ewes that exhibited vaginal bleeding or aborted were immediately euthanized with a powder-activated, penetrating captive bolt gun, as per AVMA Guidelines on Euthanasia [19]. Following immediate loss of consciousness and loss of corneal reflex, pneumothorax was created as an adjunctive method to ensure death. The study was concluded 21 days post-challenge and all remaining animals were euthanized and necropsied.

### Necropsy

At necropsy ewes and fetuses were inspected for gross lesions and samples were collected for bacterial culture, pharmacokinetics and histologic examination. Post-mortem culture samples collected from each ewe included heart blood and uterus. Fetal placenta (abortions) or intact placentome and a fetal lung/liver tissue homogenate were also harvested for bacterial culture. Samples of uterus, placenta/placentome and pooled fetal lung and liver were placed in separate sterile Petri dishes. Samples were immediately refrigerated following collection and cultured the same day. Tissues collected for pharmacokinetics included uterus, placenta/placentome, fetal amniotic fluid, lung and liver. Amniotic fluid was harvested with a 3 mL syringe and 22-gauge needle and stored in a snap-cap tube at -80 immediately following harvest. At least 30 grams of each tissue were collected in whirl-pak bags and stored at -80°C immediately following harvest. Samples collected for histopathology included maternal liver, gall bladder, and uterus. Fetal tissues collected for histologic examination included placenta/placentome, fetal lung and liver. Tissues for histopathology were placed in 10% neutral buffered formalin for 24 hours and then transferred to 70% ethanol, trimmed, and processed routinely for H&E staining. All placentas/placentomes were stained with Gimenez stain to assess for intracytoplasmic organisms consistent with *Coxiella burnetii* or chlamydia.

### *Campylobacter* culture

For *Campylobacter* culture and semi-quantitative enumeration of *C*. *jejuni* from necropsy samples, a couple drops of blood were directly streaked onto agar culture plates using a sterile cotton swab. Placenta, uterus, and pooled fetal liver and lung tissues were minced with sterile scissors or scalpels, swabbed and streaked onto media. The culture medium was Mueller-Hinton (MH) agar containing Preston *Campylobacter* selective supplement (trimethoprim, rifampicin, polymyxin B and cycloheximide; SR0117E) and *Campylobacter* growth supplement (SR0232E; sodium metabisulfite, sodium pyruvate and ferrous sulfate). Incubation took place in anaerobic jars under microaerobic conditions at 42°C for 48 hours. *Campylobacter*-like colonies were counted on each plate to determine the number of CFUs in each sample. A single suspect colony from each sample and/or animal was subjected to species identification by MALDI-TOF mass spectrometry as described elsewhere [20].

## Bioanalytical methods

### Chemicals

Solvents used in the LC-MS/MS analysis of tulathromycin were LC-MS grade (Optima, Fisher Chemical, Fair Lawn, NJ). The acetonitrile used in protein precipitation of plasma and amniotic fluid was also LC-MS grade. Analytical standards of tulathromycin and CP-60,300 were obtained from Santa Cruz Biotechnology and Toronto Research Products, respectively. The internal standards of tulathromycin-d7 and roxithromycin were obtained from Toronto Research Products and Sigma Chemical Co., respectively. Stock standards of these reference standards were prepared at a concentration of 1.00 μg/μL in LC-MS grade methanol and stored at -20°C.

#### Extraction procedures

Plasma and amniotic fluid samples were prepared by precipitation of plasma proteins with acetonitrile. Plasma samples 100 μL, were mixed with 400 μL of acetonitrile to precipitate plasma proteins. The acetonitrile contained tulathromycin-d7 as an internal standard at a concentration of 200 ng/mL. Nine calibration spikes and three quality control (QC) samples in blank ovine plasma were extracted with each set of plasma or amniotic fluid samples. The concentration of the calibration spikes was 2.5, 10, 20, 50, 100, 200, 500, 1000 and 2,000 ng/mL while the QC samples was 15, 150, and 1500 ng/mL. The samples were vortexed for 5 seconds and centrifuged for 10 minutes at 7500 rpm (6000 x g) to sediment the protein pellet. The supernatant was poured off into dry down tubes and evaporated at 40°C with a flow of nitrogen in a Turbovap. The contents were reconstituted with 125 μL of 25% acetonitrile in water followed by 75 μL of water. The samples were transferred to autosampler vials fitted with a glass insert and centrifuged at 2,400 rpm (2000 x g) prior to analysis.

Tissue samples of fetal liver, fetal lung, uterus, and placentome were extracted by acidic hydrolysis of tulathromycin to the common hydrolytic fragment, CP-60,300. Homogenized tissue samples, tissue spikes, and ovine tissue blanks, 1 gram, were hydrolysed with 2 N hydrochloric acid (HCl), 4 mL, for 1 hour at 60°C. A second addition of 3.5 mL of HCl to the tissue samples was performed after centrifugation of the tissue digest and removal of the supernatant. The samples were then vortexed and shaken followed by centrifugation. The supernatant from this second extraction was combined with the supernatant from the first digestion and the volume was adjusted to 8 mL. Each set of tissue samples was run with seven calibration spikes (tulathromycin) prepared in the corresponding blank ovine tissue matrix along with tissue blank. These calibration spikes were at concentrations of 0.02, 0.05, 0.10, 0.20, 0.50, 1.0, and 2.0 ug/g. Negative sheep plasma, uterus, placentome, amniotic fluid, fetal lung and liver, were collected from untreated sheep from a separate study. For LC-MS/MS analysis the samples and spike/blanks were diluted 1:10 with a 0.1 M potassium acetate buffer, pH 5.0 in autosampler vials. The buffer contained an internal standard of roxithromycin at a concentration of 50 ng/mL. The vials were then centrifuged at 2,400 rpm prior to analysis.

#### LC-MS/MS analysis

LC-MS/MS was performed using a Surveyor Pump and autosampler coupled to a triple quadrupole mass spectrometer TSQ Discovery Max (Thermo Scientific, San Jose, CA, USA). The mobile phases consisted of A: 0.1% formic acid in water and B: 0.1% formic acid in acetonitrile at a flow rate of 0.25 mL/min. Separation was achieved with an ACE 3 C18 column, 150 mm x 2.1 mm, 3 μm particles (Mac-Mod Analytical, Chadds Ford, PA, USA) maintained at 45°C. The autosampler temperature was 12°C with an injection volume of 15 μL. Initial solvent composition was 7.5% B which was increased linearly to 95% B in 8 minutes. The solvent composition was maintained at 95% B for 2 minutes prior to equilibration to 7.5% B. The flow rate during this time period was 0.325 ml/min. Tulathromycin and tulathromycin-d7 eluted from the ACE C18 column at 5.05 ± 0.05 minutes. Positive ion electrospray MS of the precursor ions of the analytes was used for residue detection. The triply charged precursor ions of tulathromycin (m/z 269.8) and tulathromycin-d7 (m/z 271.9) were used for MS fragmentation in the analysis. The fragment ions of the triply charged tulathromycin and tulathromycin-d7 precursors were 115.9, 259.1, and 420.1 m/z.

The tulathromycin marker, CP-60,300, and roxithromycin eluted from the ACE 3 C18 column at 4.81 ± 0.05 and 7.43 ± 0.05 minutes, respectively. Positive ion electrospray MS of the precursor ions of the analytes was used for residue detection. The doubly charged precursor ion of CP-60,300 (m/z 289.2) and singly charged roxithromycin (m/z 837.5) were used for MS fragmentation in the tulathromycin analysis. The fragment ions of the doubly charged CP-60,300 marker precursor at m/z 289.4 were 116.0, 158.1, 231.1, and 420.3 m/z. The fragment ions of the roxithromycin precursor ion at m/z of 837.5 were at 522.2, 558.3, and 679.4 m/z.

Calibration curves in plasma and amniotic fluid exhibited a correlation coefficient (r^2^) exceeding 0.995 across the concentration range using a weighted (1/X) linear fit. QC samples at 15, 150, and 1500 ng/mL were within ± 15% of the nominal value with most of the QC’s within ± 10% of the nominal value. The limit of quantitation (LOQ) of the analysis was 2.5 ng/mL with a limit of detection (LOD) of 0.5 ng/mL.

All tissue calibration curves exhibited a correlation coefficient (r^2^) exceeding 0.99 across the concentration range using a weighted (1/X) linear fit. The limit of quantitation (LOQ) of the analysis was 0.02 ug/g with a limit of detection (LOD) of 0.05 μg/g for fetal liver and fetal lung. The limit of quantitation (LOQ) of the analysis was 0.02 μg/g with a limit of detection (LOD) of 0.05 μg/g for ovine uterus and placentome. A few uterus samples were rerun after dilution with the blank uterus extract as the concentration of the sample was above the 2 μg/g level.

#### Method development and validation

Tulathromycin has been measured in three plasma matrices in this laboratory by LC-MS/MS for over six years. A simple protein precipitation with acetonitrile has always afforded a robust analysis. The LC-MS/MS analysis for tulathromycin has been performed on ion trap instruments as well as two different triple quadrupole instruments and an Orbitrap exact mass instrument. USDA regulations through the Food Safety and Inspection Service (FSIS) require analysis of CP-60,300 rather than tulathromycin in animal tissues. The hydrolytic fragment, CP-60,300, was not available commercially until two years ago at which time we switched analysis to this residue in tissues. The hydrolysis procedure with 2 N HCl is the standard FSIS method in tissues. This method has been previously validated in caprine tissues [21,22].

#### Pharmacokinetic analysis

As previously described by Smith et al., pharmacokinetic analysis of total tulathromycin plasma concentrations was completed using a statistical moment (i.e., non-compartmental) approach in commercial software (PKanalix, Monolix Suite 2019R1, Lixoft, France) [22]. Time versus concentration figures for tulathromycin were produced using a commercial program (GraphPad Prism version 7.0 for MacOS, GraphPad Software, La Jolla California USA, www.graphpad.com).

Standard PK parameters were generated for individual sheep, as follows:

- Maximum tulathromycin concentration, **Cmax**;
- Time of maximum tulathromycin concentration, **Tmax**;
- Area under tulathromycin concentration-time curve, **AUClast**, **AUCinf** and partial AUC estimate from 0 to 72 hr (**AUC0-72**);
- Area under the moment curve, **AUMCinf**;
- Tulathromycin mean residence time, **MRT**

MRT = AUMCinf ⁄ AUC inf;

- Slope of the elimination phase **λ**_**z**_, computed by linear regression of the logarithmic concentration vs. time curve during the elimination phase;
- Tulathromycin terminal half-life, **T**_**1/2**_ **(λz)**

T_1/2_ (λz) = ln (2) ⁄ λz;

- Tulathromycin apparent systemic clearance, **CL/F**

CL/F = Dose ⁄ AUC inf;

- Apparent volume of distribution of tulathromycin during the elimination phase, **Vz/F**

Vz/F = Dose ⁄ (AUCinf x λz);

For data analysis, the first value below the LLOQ was inferred to be LLOQ/2, and subsequent data points were excluded from the analysis. A linear/log trapezoidal rule was used to estimate the area under the tulathromycin time-curves.

Selection of timepoints for determination of λz for each individual was performed automatically by the PKanalix 2019R1 software using the adjusted R2 method and checked manually prior to running the non-compartmental analysis. A minimum of 3 timepoints was selected for estimating the slope of the terminal phase. λz was calculated via a linear regression between Y = log(concentrations) and the X = time. The 1/Y^2^ weighting method was used for the regression analysis.

Summary statistics on the individual PK parameters were performed thereafter to derive the geometric mean, median and (min-max) range. The geometric mean instead of the arithmetic mean was used given the small size of the study and the relatively large amount of data below the analytical quantification limit.

### Statistical analysis of tulathromycin tissue data

Graphical representations of tissue tulathromycin data (not presented herein) were performed using the ggplot2 package (v. 3.2.1) in R 3.5.2. Differences in average tissue concentration were assessed with a student’s T test. *P* < 0.05 were considered as statistically significant.

## Results

### Animals

Six hours post C. *jejuni* challenge, one ewe (C10) was laterally recumbent and had developed respiratory distress. She was euthanized 6.8 hours post-*C*. *jejuni* challenge for humane reasons. At necropsy she had abundant white foam in her trachea and nostrils. Her lungs were extremely heavy, and a white froth and clear fluid drained from large airways when the lungs were sectioned (pulmonary edema). The pleural surface of the lung had numerous petechial hemorrhages. Two fetuses were present *in utero*, each of which had multifocal petechial to ecchymotic hemorrhages in the subcutis and liver. *C*. *jejuni* was not isolated from maternal or fetal tissues. Clinical and post-mortem findings were interpreted to support a diagnosis of severe endotoxic shock.

Three of the 12 ewes (C5, C6, C12) developed vaginal bleeding or aborted. Large numbers of *C*. *jejuni* were isolated from the uterus, fetus and placenta of each of these ewes accompanied by histologic evidence of metritis and placentitis (Table 1). Results support a diagnosis of *C*. *jejuni* abortion in these three animals. A fourth ewe (C11) had a live fetus *in utero* at the 21-day study termination, but large numbers of *C*. *jejuni* were isolated from placentomes in association with mild placentomal inflammation. *C*. *jejuni* was not isolated from tissues of the remaining 6 ewes (C1-4, C7-9) and significant gross or histologic lesions were not identified in fetal or maternal tissues from these animals. Gimenez stained sections of placenta/placentome failed to reveal intracytoplasmic organisms consistent with *Coxiella burnetii* or chlamydia in any of the ewes.

**Table 1:** Histopathology and *Campylobacter jejuni* culture results on fetal and maternal tissues harvested at necropsy.following tulathromycin administration in campylobacter-challenged, pregnant ewes.

Animal ID	DPI	Vaginal bleed, Aborted	Metritis	Placentitis	Placenta	Uterus	Fetus	Maternal Blood
C1	6	-	0	0	0	0	0	0
C2	3	-	0	0	0	0	0	0
C3	11	-	0	0	0	0	0	0
C4	11	-	0	0	0	0	0	0
C5	5	+	+	+	Lawn	Lawn	TNTC	0
C6	3	+	+	+	Lawn	Lawn	TNTC	0
C7	21	-	0	0	0	0	0	0
C8	3	-	0	0	0	0	0	0
C9	21	-	0	0	0	0	0	0
C10	1	Endotoxic shock	0	0	0	0	0	0
C11	21	-	0	+	TNTC	0	0	0
C12	8	+	+	+	TNTC	TNTC	TNTC	0

Lawn: Culture plate completely covered with bacterial colonies.

TNTC; Too numerous to count.

DPI: Number of days post-campylobacter challenge the animal was euthanized.

### Non-compartmental analysis

**Fig 1:**
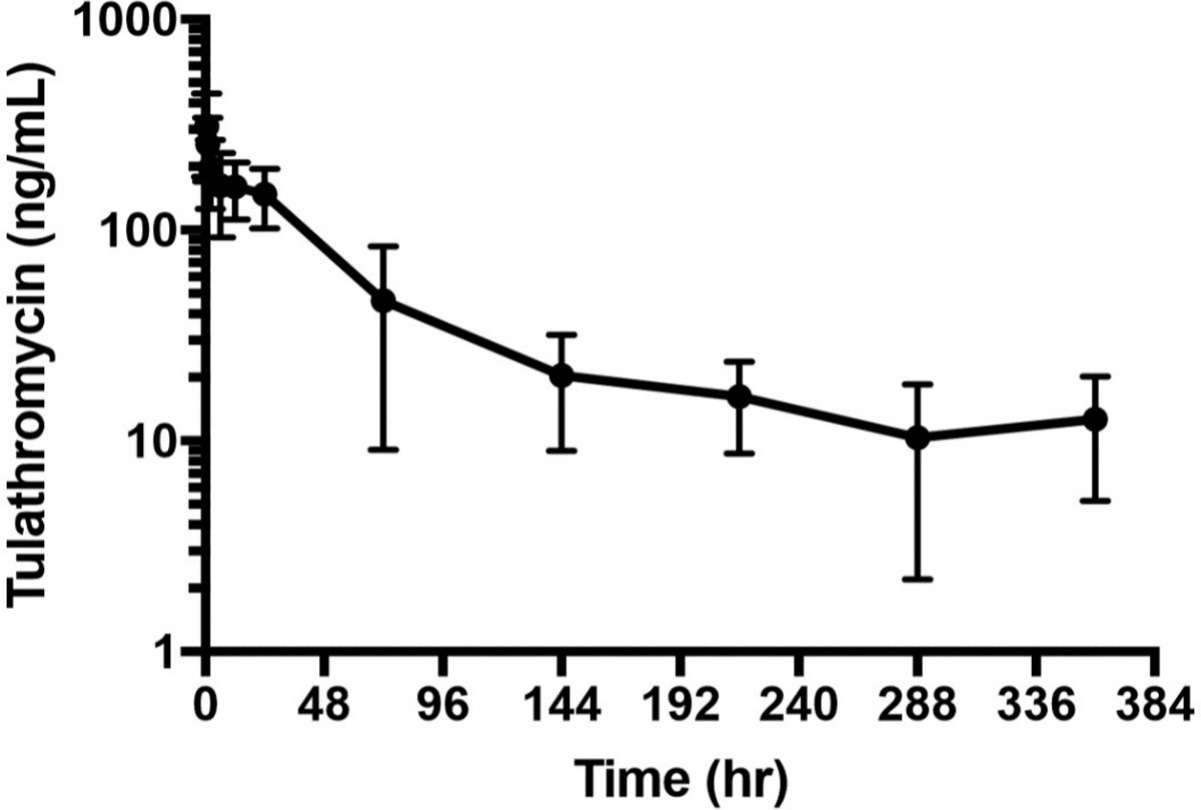
Plasma tulathromycin concentrations (ng/ml) over time (hours). Data are presented on a log10 scale with mean and one standard deviation.

No sheep had detectable tulathromycin in plasma at time zero. Geometric mean and standard deviations disposition profiles are presented in Table 2. There appeared to be significant variations of time versus concentration data for tulathromycin among individual sheep. For an LLOQ of 2.5 ng/mL, 26% (38/144) of the samples had values below the analytical quantification limit. Consequently, the number of samples above the quantification limit was highly variable among study subjects, with some individuals only presenting 3 measurable concentrations of tulathromycin in plasma (i.e., Ewe C10). The AUC% extrapolation was estimated to be inferior to 20%. The plasma pharmacokinetic parameters for tulathromycin when administered subcutaneously are summarized in Fig 1. Reported below are the plasma (geometric) mean pharmacokinetic parameters with (min-max) range values for tulathromycin in pregnant ewes challenged with *Campylobacter jejuni*: the maximum tulathromycin plasma concentration was estimated at 302.0 (153.9–553.5) ng/mL, with a peak level observed at around 1.2 (0.5–24.0) hours. The apparent systemic clearance of tulathromycin was estimated at 16.6 (4.5–217.6) L/h (or 0.28 L/kg/h), associated with a low global extraction ratio (E = 0.06), calculated as CL/Qc (with cardiac output Qc (mL/kg/min) approximated by the formula: Qc = 180*BW^(-0.19)^ [23]. Lastly, the apparent steady-state volume of distribution of tulathromycin in sheep was large, 522.0 (184.9–2879.1) L (or 9.0 L/kg), with an elimination half-life estimated at approximately 22 hours.

**Table 2:** Summary results from the non-compartmental analysis (NCA) of plasma tulathromycin data in 12 pregnant ewes challenged with *Campylobacter jejuni*.

	Units	Min	Median	Max	Geomean	GeoSD
**C**_**max**_	ng/mL	153.9	302.1	553.5	301.5	1.5
**T**_**max**_	hr	0.5	0.5	24.0	1.2	3.8
**AUC**_**last**_	ng/mL x hr	564.3	10,419.9	20,080.7	7,956.4	2.6
**AUC**_**inf**_	ng/mL x hr	565.3	10,465.4	33,812.8	8,477.0	2.8
**AUC**_**(0–72)**_	ng/mL x hr	605.5	7,453.6	13,230.9	6,088.5	2.2
**AUMC**_**inf**_	ng/mL x hr^2^	852.5	637,544.2	15,761,900.0	358,667.6	11.3
**MRT**	hr	1.5	48.9	466.2	42.3	4.2
**λz**	1/hr	0.002	0.04	1.18	0.03	5.5
**T**_**1/2**_ **(λz)**	hr	0.6	18.3	446.9	21.8	5.5
**CL/F**	L/hr	4.5	14.6	217.6	16.6	2.7
**Vz/F**	L	184.9	373.8	2879.1	521.5	2.4

^1^Maximum plasma concentration.

^2^Time of maximum plasma concentration.

^3^Area under the concentration-time curve from time 0 to the last observable timepoint.

^4^Area under the concentration-time curve from time 0 to infinity.

^5^Area under the concentration-time curve from time 0 to 72 hours.

^6^Area under the moment curve from time 0 to infinity.

^7^Mean residence time.

^8^Slope of the terminal (elimination) phase.

^9^Terminal (elimination) half-life.

^10^Apparent systemic clearance.

^11^Apparent volume of distribution during the elimination phase.

Several pharmacokinetic parameters were produced via NCA using Pkanalix 2019R1 (Lixoft, France). The following summary parameters are reported for Tulathromycin: **Cmax**^1^; **Tmax**^2^; **AUClast**^3^, **AUCinf**^4^ and **AUC0-72**^5^; **AUMCinf**^6^; **MRT**^7^; **λ**_**z**_^8^; **T**_**1/2**_ **(λz)** ^9^; **CL/F**^10^; and **Vz/F**^11^.

### Tulathromycin tissue concentrations

A summary of tulathromycin tissue concentrations in pregnant ewes is provided in Table 3. The mean tissue concentrations were highest in the uterus (2.464 μg/g), followed by the placentome (0.484), fetal liver (0.11) and fetal lung (0.03). The mean concentration of Tulathromycin detected in the uterus of ewes with high levels of Campylobacter in the reproductive tract and conceptus were compared to ewes that were culture negative at necropsy. The mean concentration of Tulathromycin was 1.745 μg/g ± 1.04 in infected and inflamed uteri and 2.82 μg/g ± 1.24 in non-infected uteri. Due in part to the high individual animal variation, these differences were not statistically significant.

**Table 3:** Tulathromycin concentrations in selected maternal and fetal tissues at various time periods following antibiotic administration in campylobacter-challenged, pregnant ewes.

Animal ID	Necropsy–hours post-antibiotic administration	Campy infection (i.e., culture positive)	Placentome (μg/g)	Uterus (μg/g)	Amniotic fluid (μg/ml)	Fetal liver (μg/g)	Fetal lung (μg/g)
C10	7	Neg	0.20	4.03	0.0051	0.04	0.02
C8	50	Neg	0.56	2.33	0.0023	0.07	0.02
C2	50	Neg	0.59	3.93	0.0028	0.11	0.03
C6	56	Pos	0.58	1.16	NA	0.06	0.02
C5	105	Pos	0.40	0.99	------	0.19	0.12
C1	120	Neg	0.48	1.14	0.0025	0.07	0.02
C12	166	Pos	0.48	1.57	0.0372	0.09	0.04
C4	239	Neg	0.55	4.18	0.0018	0.10	0.03
C3	240	Neg	0.51	2.84	0.0025	0.11	0.02
C9	478	Neg	0.48	1.06	0.0025	0.13	0.03
C7	478	Neg	0.37	3.08	0.0077	0.19	0.02
C11	479	Pos	0.61	3.26	0.002	0.06	0.03
Mean ± SD			0.48 ± 0.11	2.464 ± 1.19	0.0066 ± 0.011	0.101 ± 0.049	0.03 ± 0.028

The amniotic fluid results of C5 were not reported because they were a statistical outlier.

## Discussion

Our analysis exclusively focused on the pharmacokinetics of unchanged tulathromycin. Pharmacokinetic studies in ruminants and pigs have shown that tulathromycin is metabolized to a low extent and is eliminated primarily as the unchanged (parent) drug (EMEA/MRL/894/04). As a result, very low concentrations of tulathromycin metabolites are typically detected in plasma. Therefore, most pharmacokinetic studies from the literature focus on unchanged tulathromycin. When the plasma pharmacokinetic dispositions of tulathromycin from this study were compared with data from similar studies using the same dose, formulation, schedule and route, in non-challenged pregnant sheep and non-pregnant adult ewes, the mean maximum plasma tulathromycin concentration in *C*. *jejuni* challenged sheep was much lower (0.3 μg/mL) than in pregnant sheep (4.9 μg/mL) and non-pregnant ewes (3.6 μg/mL), the mean time to maximum concentration was shorter (1.2 hrs) when compared to pregnant sheep (4.0 hrs) but similar to non-pregnant adult ewes (1.6 hrs), and the mean apparent elimination half‐life was substantially shorter (22 hrs) than in pregnant sheep (110.8 hrs) and non-pregnant ewes (118 hrs) [11,13]. The differences in maximum plasma tulathromycin concentration have been observed in other experimental models of infection, with lower concentrations observed in goats and pigs undergoing experimental respiratory infection compared to controls [22,24]. It is possible that the leukocyte transport of tulathromycin to sites of infection decreased the plasma concentrations of the ewes in this study. Additionally, volume of distribution can be altered by different physiologic states, such as pregnancy, with absorption typically decreased and elimination increased in pregnant individuals [25]. Due to high tissue distribution of macrolides such as such tulathromycin, such disparate Cmax levels in plasma in animals of varying physiological state may not be indicative of differences in clinical efficacy.

Using the standard equations outlined in the Methods section (*Pharmacokinetic analysis*), we calculated the apparent systemic clearance (CL/F) and volume of distribution (Vz/F) of tulathromycin based on earlier descriptions from the literature [11,13]. Specifically, CL/F after a single subcutaneous dose of tulathromycin (2.5 mg/kg) was estimated at 10.8 mL/kg/h and 29.1 mL/kg/h in non-challenged pregnant sheep and non-pregnant adult ewes, respectively [11,13]. In comparison, the estimated apparent systemic clearance of tulathromycin in *C*. *jejuni* challenged sheep was much higher (280 mL/kg/h). The apparent volume of distribution of tulathromycin was estimated at 1.54 L/kg and 4.05 L/kg in non-challenged pregnant sheep and non-pregnant adult ewes, respectively [11,13]. Comparatively, the estimated Vz/F in *C*. *jejuni* challenged sheep was 9.0 L/kg.

This study utilized an aggressive IV challenge model that typically results in loss of approximately 10% of challenged ewes due to endotoxic shock (in house, unpublished data). One ewe from this study was euthanized in extremis ≈ 6 hours post-challenge with clinical signs and gross lesions consistent with endotoxic shock. The LPS constituent of the outer membrane of Gram-negative bacteria such as campylobacter has been linked to inflammation and immune activation in a wide range of pathologies [26]. It appears that endotoxemia associated with IV administration of large numbers of *C*. *jejuni* may alter the pharmacokinetics of tulathromycin. However, this aggressive challenge model may not accurately reflect field cases of abortion where clinical signs in ewes are not typically reported prior to abortion.

Sepsis is characterized by a state of increased vascular permeability responsible for a shift of fluids from the intravascular compartment to the interstitial space [27,28]. The systemic effects associated with IV administration of Campylobacter, a gram-negative bacterium, could be responsible for an increase of tulathromycin volume of distribution in *C*. *jejuni* challenged sheep. Our findings on tulathromycin apparent clearance are more surprising since earlier descriptions on the effect of infectious and inflammatory diseases on cytochrome P450-mediated drug metabolism and pharmacokinetics have reported down-regulations of hepatic and extrahepatic cytochrome P450s, as well as other drug metabolizing enzymes [29]. Noteworthily, any change in the apparent clearance and volume of tulathromycin can be confounded by variations in subcutaneous bioavailability. Although the bioavailability of tulathromycin after intramuscular administration in sheep has been reported at 100% (Draxxin Summary of Product Characteristics), to the best of the authors knowledge, its value after subcutaneous dosing in unknown. In essence, our reported changes in apparent clearance and volume can be triggered by a change (i.e., reduction) in subcutaneous bioavailability.

Overall, results of this study demonstrate that prior IV administration of *C*. *jejuni* appears to substantially alter the pharmacokinetics of tulathromycin, reducing both the peak plasma concentrations and elimination half-life. Additional studies utilizing a control group consisting of pregnant ewes not challenged with campylobacter would be needed to definitively confirm these suspicions. Furthermore, although the sampling schedule in our study was quite dense, parameter estimates derived from a non-compartmental analysis are heavily dependent on selected sampling times. Additionally, the large variation in the number of quantifiable samples of tulathromycin in plasma among study subjects likely drove a significant part of the variability in pharmacokinetic parameter estimates between ewes. As such, additional mathematical modeling work on a larger study population is warranted to verify our preliminary findings on the effect of *C*. *jejuni* on tulathromycin pharmacokinetics in sheep.

In this study, ewes were pretreated with flunixin meglumine, which is a non-steroidal anti-inflammatory used in the treatment of inflammatory conditions, including endotoxemia [30]. Previous unpublished studies in our laboratory have demonstrated that flunixin meglumine pretreatment diminishes the number of ewes that succumb to endotoxemia within the first 24 hours following IV administration of campylobacter. This drug inhibits cyclooxygenase thereby decreasing prostaglandin synthesis [31,32]. In one report in sheep, flunixin meglumine slowed the elimination and increased plasma concentrations of levofloxacin [31]. It was speculated that inhibition of prostaglandin synthesis in the kidneys by flunixin meglumine caused reduced renal blood flow and a reduced glomerular filtration rate leading to decreased excretion of the antibiotic via urine [31]. One study in goats did demonstrate that when flunixin meglumine was administered concurrently with tulathromycin the pharmacokinetics were altered [33]. Given that the present study is done in a different species and that the drugs were not administered simultaneously it is difficult to speculate on what impact this might have in the present study. Future studies to further evaluate this issue are warranted.

The average tissue tulathromycin concentrations were highest in the uterus (2.46 μg/g) and placentome (0.48 μg/g) and lowest in fetal liver (0.11 μg/g) and lung (0.03 μg/g). Since the first tissue samples were harvested 48 hours post-tulathromycin treatment, it is unlikely that this study identified peak tissue concentrations. Results do demonstrate that during the 18-day tissue sampling period, the decay of tulathromycin in tissues was very prolonged with similar levels detected at Days 2 and 20 post-treatment in all tissues and fetal fluids. These persistent levels in the uterus, placentome, amniotic fluid, fetal lung and liver for many days beyond administration of a single dose of tulathromycin indicates therapeutic potential. Additional studies with necropsies at earlier time periods would be needed to determine peak concentrations in these tissues. Of note, in this study tulathromycin was administered 18 hours post-*Campylobacter* challenge. During outbreaks of *Campylobacter* abortion, ewes will have been exposed at different time periods. Currently a paucity of information exists regarding the tulathromycin concentrations in the lungs of sheep with lower respiratory tract infections. In other species, lung concentrations have reached levels of 3.47 μg/g (pigs, 24 hours post infection), and 5.3 μg/mL in the pulmonary epithelial lining fluid in calves [34,35]. These levels are similar to the concentrations achieved in the uteruses of the ewes in our study. Susceptibility results on C. jejuni cultured from field cases of ovine abortion have demonstrated that the vast majority of isolates are likely susceptible to the macrolide antibiotics (including tulathromycin, azithromycin, telithromycin and erythromycin), based on comparable breakpoints for C. jejuni to macrolide antibiotics established in other species [1,8,9]. Administering tulathromycin at various times post-*Campylobacter* challenge would provide additional information on whether the persistent tissue levels are sufficient to eliminate infection from the uterus and conceptus in animals following various exposure intervals.

An interesting observation was that the ewes with generally lower reproductive tissue tulathromycin concentrations had the more obvious signs of infection. While the small sample size limits the statistical evaluation of this relationship, it is possible that lower tissue concentrations of tulathromycin aided establishment of infection. Macrolides are known to concentrate at the site of infection due to transport by leukocytes, although this relationship is best described in pulmonary tissues [24,36,37]. Future studies will need to evaluate the effect of low tissue concentrations on infection in the reproductive tissues, as well as the effect of reproductive infections on the distribution of macrolide antibiotics.

Importantly, the administration of tulathromycin to treat pregnant ewes during an abortion storm is an extra-label use of this antibiotic and requires a valid veterinarian-client-patient relationship and appropriate veterinary oversight. In the United States or Canada, veterinarians could contact the Food Animal Residue Avoidance Databank (FARAD), or Canadian Global Food Animal Residue Avoidance Databank (CGFARAD) for extra-label use withdrawal period recommendations. Veterinarians should evaluate available diagnostic information and consider prudent use principles when determining if tulathromycin is an appropriate therapy. This study was not designed to evaluate, nor was it intended to advocate, the use of tulathromycin as a metaphylatic treatment, as this practice may facilitate the development antibiotic resistance [38,39].

Conclusions: Overall, this study illustrates that disease states, including endotoxemia and the inflammation associated with campylobacter infection, have the potential to impact the pharmacokinetics of tulathromycin in pregnant ewes. Primarily, volume of distribution and clearance are increased compared to studies evaluating the pharmacokinetics in non-infected ewes. Additionally, the administration to infected ewes results in decreased peak concentrations and a reduced half-life when compared to healthy ewe studies. Uterine tissues maintained the highest concentrations of tulathromycin when compared to placentomes, amniotic fluid, as well as fetal liver and lung. While the persistence of tulathromycin in tissues post infection is promising, ewes in this study with lower reproductive tissue concentrations appear to have had the more severe clinical disease. The decreased peak concentrations and shortened half-life in challenged animals would suggest that more than one tulathromycin injection may be required to diminish losses during a *C*. *jejuni* abortion storm.

## Abbreviations

CFU: colony forming units
IV: intravenous
*C. jejuni*: *Campylobacter jejuni*
